# Anterior Cruciate Ligament Injury Prevention Program Implementation for High School Athletes Is Extremely Low and Faces Surmountable Barriers

**DOI:** 10.1002/ars2.70037

**Published:** 2026-06-08

**Authors:** Hannah R. Popper, Katherine Jerakis, Emily Schmeiser, Richa Gupta, Kevin B. Freedman, Steven Cohen, Meghan E. Bishop

**Affiliations:** ^1^ Jefferson Health New Jersey Stratford New Jersey U.S.A.; ^2^ Drexel University College of Medicine Philadelphia Pennsylvania U.S.A.; ^3^ Philadelphia College of Osteopathic Medicine Philadelphia Pennsylvania U.S.A.; ^4^ Perelman School of Medicine at the University of Pennsylvania Philadelphia Pennsylvania U.S.A.; ^5^ Rothman Orthopaedic Institute Philadelphia Pennsylvania U.S.A.

## Abstract

**Purpose:**

To assess the rates of usage of anterior cruciate ligament injury prevention programs (AIPPs) among high school athletes and identify barriers to implementation.

**Methods:**

After Institutional Review Board exemption, high school sports teams covered by a regional multispecialty orthopaedic practice were identified. Athletic directors, trainers, and coaches for each school were contacted for completion of a survey on (1) sex, level, size, and coaching/training staff of each team; (2) incidence of anterior cruciate ligament injuries among players over the past 5 years; (3) team's use of AIPPs; and (4) interest and perceived barriers related to AIPP use.

**Results:**

Thirty‐eight representatives from 522 teams (54.0% boys’ teams, 46.0% girls’ teams) across 22 high schools responded to the survey. Eighteen teams (3.5%) were found to use AIPPs. The 27 (87.1%) of the 31 representatives from the remaining 504 teams reported being aware of the existence of AIPPs. The most common barrier reported against implementing AIPPs was a lack of trained staff (43.6%), followed by time constraints (33.0%).

**Conclusions:**

Despite widespread knowledge of AIPPs across high school athletic team coaching and training staff, only 3.5% of teams currently utilize these programs. Most common barriers to implementing these programs include a lack of trained staff and time restraints during training.

**Clinical Relevance:**

Numerous AIPPs show promising results, but their adoption remains inconsistent. It is important to evaluate the awareness and implementation of AIPPs among coaches and trainers and investigate barriers to their adoption. Information from this study may help increase the use of AIPPs and thereby reduce the incidence of anterior cruciate ligament injuries in young athletes.

## Graphical abstract

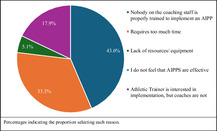

Injury to the anterior cruciate ligament (ACL) is one of the most common knee injuries, with a reported incidence of approximately 100,000 cases annually in the USA.^1^ Around 70% of ACL injuries occur as a result of sports participation, often due to noncontact mechanisms including sudden changes in direction, jumping, and cutting movements.^1^, ^2^ Surgical intervention is considered widely successful for these injuries, with more than 90% of young athletes returning to sports participation and approximately 78% returning to preinjury or higher levels of sport.^3^ Nevertheless, ACL injuries remain significantly debilitating for athletes, typically requiring 6 to 12 months of rehabilitation for full recovery.^1^ Given this high incidence of injury, there has been considerable interest in developing injury prevention strategies. Numerous ACL injury prevention programs (AIPPs) have been established to address the neuromuscular imbalances and biomechanical risk factors commonly associated with ACL injury. These programs consist of drills and techniques aimed at strengthening the hamstrings, hips, core, and trunk, overall helping to reduce asymmetry and ground reaction forces during landing, jumping, cutting, and twisting movements.^2^, ^4^, ^5^, ^6^ Among the established AIPPs, fédération internationale de football association 11+, Prevent Injury and Enhance Performance, and Sportsmetrics are considered the most widely adopted.^2^

Furthermore, AIPP programs, including Knee Injury Prevention Program (KIPP) and Sportsmetrics, have been specifically tailored for female athletes,^5^ who are reported to be up to 8 times more likely to sustain ACL tears compared with males.^7^ A 2018 meta‐analysis examining the effectiveness of AIPPs reported that they reduce the risk of noncontact ACL injuries by an average of 50% in athletes overall and by 67% on average in female athletes, confirming the benefit of usage.^8^

Despite promising results, AIPP adoption remains inconsistent, and ACL injury rates remain high.^1^ A 2022 review by Arundale et al.^9^ identified several barriers to AIPP usage, including cost, time constraints, and negative perceptions of AIPPs as “boring” or ineffective. When focusing on implementation among high‐risk sports such as soccer and basketball, factors such as lack of experienced staff, time constraints during practice, and similar activities to AIPPs are already being performed during practice affect the adoption of prevention programs among youth sports teams.^10^, ^11^ Implementation delays are also common, with widespread usage among other prevention programs taking up to 17 years.^12^ Numerous solutions have been proposed to mitigate these barriers, but current evidence remains limited, primarily based on single‐team, single‐sport, and single‐gender studies.^13^, ^14^

The purpose of this study was to assess the rates of usage of AIPPs among high school athletes and identify barriers to implementation. It was hypothesized that increased awareness and increased ACL injury rates are associated with increased AIPP utilization among teams.

## METHODS

After Institutional Review Board exemption was obtained, male and female high school sports teams covered by a large regional multispecialty orthopaedic practice of the greater Philadelphia area as of June 2023 were identified. Internal contact information and online searches were used to collect contact information for each school's athletic director. A cross‐sectional survey study was performed using a REDCap survey. A REDCap survey created by the authors of this study was sent to the athletic director of each high school along with a description of the study and a request to distribute the survey to a representative (athletic trainer or coach) for each boys’ and girls’ sports team at their school. It was specified that the response to this survey was completely voluntary and would not affect the quality of their orthopaedic team coverage.

The survey included original items that were created by the authors of this study. Items included in the survey collected information on the (1) sex, level, size, and coaching/training team of each sports team available at the school; (2) estimated incidence of ACL injuries for players of each team over the past 5 years; (3) use of AIPP for each sports team; and (4) motivations, interest, and perceived barriers related to using an AIPP. Each representative responding to the survey was asked for the name of their affiliated high school to ensure duplicate responses for the same teams were not analyzed in this study. These school names were eliminated, and responses were deidentified following this review.

Representatives for each team were contacted a total of 3 times via phone and/or email for completion of the survey, with at least a week in between each contact attempt. Nonresponsive teams were not included in the results of this study nor included in the statistical analysis. T‐tests and descriptive statistics were completed using SigmaStat 4.0 (Systat, San Jose, CA) and Microsoft Excel (Microsoft, 2018) for team‐level and sex groups.

## RESULTS

### Team Demographics

The authors contacted a total of 53 unique high schools in the specified network. A total of 38 respondents across 22 unique high schools answered the survey, resulting in a response rate of 41.5%. These 38 respondents provided survey responses for a total of 522 unique boys’ and girls’ sports teams. Twelve of these representatives were team coaches, 20 were athletic trainers, and 6 were athletic directors. Of the teams that were represented, 54.0% (282 teams) were boys’ teams, and 46.0% (240 teams) were girls’ teams. Further stratification of the teams is shown in Table 1.

**TABLE 1:** Number of High School Sports Teams Represented in This Study, Stratified by Sport, Sex, and Level of Play

Varsity Teams				Junior Varsity Teams			
	Total	Boys	Girls		Total	Boys	Girls
Baseball	19	19		Baseball	19	19	
Basketball	36	19	17	Basketball	36	19	17
Cheer	6		6	Cheer	5		5
Field hockey	19	1	18	Field Hockey	17	1	16
Football	23	23		Football	18	18	
Golf	4	2	2	Golf	4	2	2
Gymnastics	2	1	1	Gymnastics	2	1	1
Hockey	5	4	1	Hockey	2	1	1
Lacrosse	30	15	15	Lacrosse	27	14	13
Soccer	38	19	19	Soccer	31	16	15
Softball	12		12	Softball	13		13
Squash	4	2	2	Squash	0	0	0
Tennis	30	17	13	Tennis	28	15	13
Volleyball	25	11	14	Volleyball	23	10	13
Wrestling	26	19	7	Wrestling	18	14	4
**Total**	**279**	**152**	**127**	**Total**	**243**	**130**	**113**

### ACL Injury History

Across teams from all sports, 18.6% (97 teams) reported having at least 1 athlete who sustained an ACL injury on their team within the past 5 years. Of these teams, 59 were boys’ teams, whereas 38 were girls’ teams. Seventy‐four of these teams were varsity teams, and 23 were junior varsity (JV) teams. Across all boys’ sports, the majority of these teams with a history of ACL injury were football teams, followed by soccer and lacrosse. Across the girls’ sports, the majority of teams were soccer and lacrosse.

### AIPP Usage

Of the 522 teams included in this study, only 3.5% (18 teams) from 6 unique high schools reported implementing an AIPP. Among these teams, 8 were girls’ teams across 4 unique high schools, and 10 were boys’ teams from 3 unique high schools. Of the boys’ teams, the sports that most frequently reported utilizing an AIPP were squash, followed by soccer and football, and then basketball, lacrosse, and tennis. Of the girls’ teams, the sports that most frequently reported utilizing an AIPP were soccer and lacrosse, followed by softball. Thirteen of the 18 teams were varsity teams, and 5 were JV teams. The distribution of the most frequently used AIPPs is described in Table 2. Furthermore, out of these 18 teams, 12 reported at least 1 ACL injury in the past 5 years. Three teams reported no ACL injuries in the past 5 years, and 3 teams reported being unsure. The sample size in this cohort was insufficient to perform further statistical comparisons on the impact of past ACL injury on the implementation of AIPPs.

**TABLE 2:** Distribution of ACL Injury Prevention Programs Utilized by Teams

	Sportsmetrics	PEP Program	KIPP	FIFA 11+	Other	Total
Teams	0/0/0	5/4/1	2/1/1	2/2/0	9/3/6	18/10/8
Percent	0%	28%	11%	11%	50%	

*Note*: Team numbers are reported as [Total number of teams using AIPP/Number of boys’ teams using AIPP/Number of girls’ teams using AIPP]. “Other” AIPPs are categorized as those with only 1 unique team in the cohort using them.

ACL, anterior cruciate ligament; AIPP, anterior cruciate ligament injury prevention program; FIFA, fédération internationale de football association; KIPP, Knee Injury Prevention Program; PEP; Prevent Injury and Enhance Performance.

The remaining 504 teams in this study were not implementing an AIPP. These 504 teams were represented by 31 survey respondents, of whom 4 reported being unaware of the existence of AIPPs, whereas 27 reported being aware of the existence of AIPPs. These 27 respondents represented 95.6% (496/522) of teams across 16 unique high schools included in this study.

All survey respondents who reported not using an AIPP for their teams were asked further questions about their interest in future AIPP education and implementation. Responses showed that 77.4% (24/31) of these team representatives reported interest in undergoing education to learn more about implementing AIPPs. Additionally, 67.7% (21/31) of team representatives reported being currently interested in implementing AIPPs into their athletes’ training/practices. More specifically, respondents were asked to rate their interest for both questions on a 7‐point Likert scale ranging from “not at all interested” to “very strongly interested”. Responses revealed an average score of 4.82 ± 1.9 for interest in undergoing education and 4.89 ± 1.5 for interest in implementing AIPPs, with a score of 4 representing a neutral interest level. Statistical comparisons were performed to ascertain if there were differences in interest levels of undergoing education and of implementing programs for representatives covering varsity versus JV level of play, as well as covering girls’ versus boys’ teams. Results from this analysis showed that interest level for undergoing education was higher for representatives covering any varsity team, with an average score of 5.63 ± 1.2, compared with representatives covering a JV team only, with an average score of 4.76 ± 1.0. Similarly, interest level for future implementation of AIPPs was higher for varsity teams, with an average of 5.54 ± 1.0, compared with representatives covering JV teams only, with an average score of 4.71 ± 1.1. Interest level scores for girls’ teams versus boys’ teams are represented in Table 3.

**TABLE 3:** Interest Scores for Undergoing Education Regarding AIPPs and for Implementing AIPPs in the Future for Both Girls’ Teams and Boys’ Teams

	Interest in Undergoing Education Regarding AIPPs	Interest in Implementing AIPPs in the Future
Girls’ teams	4.68 ± 1.6	4.70 ± 1.5
Boys’ teams	4.59 ± 1.7	4.47 ± 1.7
*P* value	.970	.921

AIPP, anterior cruciate ligament injury prevention program.

### Barriers to Using AIPPs

**FIGURE 1:**
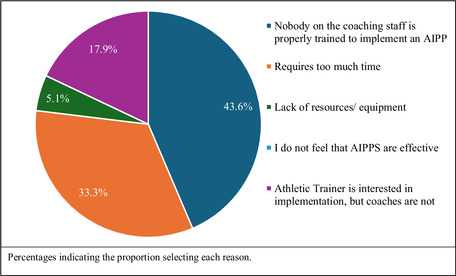
Barriers to AIPP implementation reported by study representatives. (AIPP, anterior cruciate ligament injury prevention program.)

The reported barriers from the representatives are shown in Figure 1. The most common perceived barrier is a lack of sufficient training on the coaching staff to properly implement an AIPP, selected by 43.6% (17/38) of respondents. Closely following was the perceived time constraint of implementing an AIPP (33.3%, 13/38 of respondents). Seven (17.9%) representatives chose to submit a free response with the additional message that athletic trainers for their team are interested in/have attempted to implement an AIPP, but the coaches on the team do not support these efforts, thus preventing any progress (Figure 1).

Teams that implemented AIPPs have significantly more coaching/ancillary staff compared with teams that do not implement AIPPs (4.7 ± 0.6 individuals for teams implementing AIPPs compared with 2.3 ± 1.7 individuals for teams not implementing AIPPs, *P* < .001). Additionally, AIPP‐employing teams more commonly had 3 or more different types of coaches/ancillary staff compared with teams that reported not using an AIPP. Within the cohort of teams that reported awareness of AIPPs but not their use, representatives that reported “lacking the necessary resources/ equipment” and “time constraint” as barriers to implementing an AIPP had teams with a significantly greater average athlete: staff ratio compared with those that did not select these barriers (3.77:1 vs 2.50:1, *P* = .041).

## DISCUSSION

The most important findings of this study show that usage of AIPPs remains low, with only 3.5% of high school sports teams in this study population reporting using these programs. This low rate of implementation underscores the gap between the proven effectiveness of AIPPs and their real‐world application, especially among younger, JV, and female athletes.

McFarlane et al.^15^ conducted a similar investigation in 2023 within a single section of the New York Public High School Athletic Association, reporting that although only 73% of coaches were aware of AIPPs, 52.4% implemented them into routine practice for their athletes. In contrast, findings from our current study reveal that representatives of over 90% of teams were aware of AIPPs. This could be attributed to the inclusion of athletic trainers and athletic directors in our study population as well as increased advertising efforts from AIPP organizations in the time between the 2 studies. Additionally, the current study reveals a lower rate of AIPP usage, which could be attributed to the inclusion of multiple sports, levels of play, and regional differences in AIPP adoption throughout the country. This is supported by other studies investigating AIPP use across a variety of sports, regions, levels of play, and other demographics, reporting low rates of AIPP usage ranging between 13% and 21%.^10^, ^11^, ^16^, ^17^ Thus, findings from this study could be valuable for investigating factors influencing AIPP usage and for informing future recommendations regarding AIPP adoption and education.

Further analysis into AIPP usage in this study revealed that two‐thirds (12/18) of the teams in this study that implemented AIPPs reported an ACL injury on the team within the last 5 years, suggesting heightened AIPP awareness and usage due to injury history. Additionally, our results revealed significant disparities in AIPP usage and interest in JV teams compared with varsity teams. Existing research suggests that early initiation of AIPPs during adolescent years can further increase their effectiveness and result in even greater reductions in ACL injury rates.^8^, ^18^ Thus, targeted efforts regarding AIPP use toward JV coaching staff/athletic trainers could help reduce ACL injuries among adolescent athletes. It is also important to promote targeted efforts toward adolescent female athletic teams, as this is a particularly vulnerable population to ACL injuries.^17^

The most common barriers to AIPP use identified in this study were a lack of training, followed closely by time constraints. MacFarlane et al.^15^ similarly reported that most coaches cited lack of training and resources as barriers. Time constraints have also been a concern in other studies; for example, Petersen et al.^19^ surveyed coaches of European female handball teams and reported that coaches felt prevention program exercises take away from training time for their athletes. This concern is valid, as some AIPPs recommend training sessions multiple times per week, with each session lasting over an hour.^20^ However, various strategies have successfully addressed this barrier, including incorporating AIPP exercises and drills into warm‐ups^9^, ^21^ or distributing AIPP exercises throughout practice sessions.^9^, ^13^ Many AIPPs have also developed time‐efficient alternatives, such as condensed warm‐up courses for prepractice or pregame usage, providing practical solutions for teams looking to implement AIPPs without compromising training time. Additionally, to make education of AIPPs more accessible, promoting free online access to training and time‐sensitive programs would be beneficial to encourage more widespread use of these programs. Results from this study also identified a lack of coach support as another barrier to AIPP usage. A 2021 study by Dix et al.^22^ further highlighted this barrier, reporting that over 90% of collegiate women's soccer coaches believe AIPP implementation is the responsibility of ancillary staff or even the athletes themselves. Thus, findings suggest that incorporating methods for time management and targeted education surrounding cooperation between coaches and ancillary staff into future AIPP education could mitigate key barriers associated with their use. In addition, mandatory targeted education for coaches, ancillary staff, and athletes on injury prevention programs and ways to easily incorporate exercises into scheduled practice may help increase adoption among the study's population. However, it should be noted that how involved the athletic trainers are within schools (e.g., part‐time vs full‐time), may affect ACL injury risk and institution of AIPPs.

**FIGURE 2:**
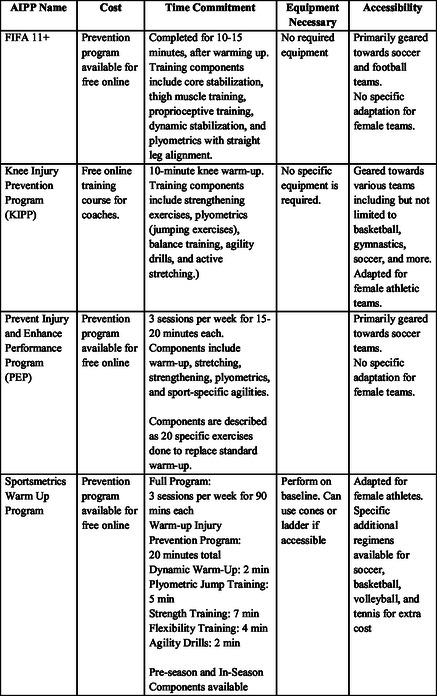
Comparison of cost, time commitment, required equipment, and accessibility among different AIPPs. (AIPPs, anterior cruciate ligament injury prevention programs; KIPP, Knee Injury Prevention Program; PEP, Prevent Injury and Enhance Performance.)

Responses from our study also revealed a wide distribution of AIPPs used among teams. In 2018, the American Academy of Orthopaedic Surgeons issued a media release^5^ stating that out of 36 AIPPs available, only 3 programs (Sportsmetrics, the Prevent Injury and Enhance Performance Program, and the KIPP) have shown clear efficacy in reducing injury incidence. Among these, only 2 (KIPP and Sportsmetrics) have been specifically tailored to the needs of female athletes. Our results revealed that a minority of teams using AIPPs use those recommended by the American Academy of Orthopaedic Surgeons, and only 1/8 of the girls’ teams using AIPPs report using KIPP or Sportsmetrics. Although the current study did not explore how teams using AIPPs selected a specific program, other studies have highlighted factors such as cost and ease of use in terms of not needing specialized equipment and time commitment.^10^, ^11^ Figure 2 provides an overview of the resources necessary for the AIPPs most mentioned in this study, revealing that they are all available for free for athletes/coaches. Given the findings of this study, the authors propose that it would be beneficial for future education of AIPPs to include specific mention of the American Academy of Orthopaedic Surgeons recommended programs, supported by comparable resources to implement these specific programs.

### Limitations

This study has several limitations. The primary limitation was the response rate; only 22 out of 53 schools provided at least 1 representative response to the survey after 3 attempts of contact, limiting the generalizability of AIPP use across a broader cohort. Additionally, the survey design allowed 1 representative to respond for multiple teams within their coverage. Although this increased team representation in this study, it reduced the ability to delineate between individual team characteristics. Since this study relied on self‐reported data from team representatives, it introduces a potential for self‐report bias by utilizing responses from only 1 representative. This study also focused on schools within a single geographic region, limiting the ability to generalize findings to other areas. In addition, there were no inquiries on whether student‐athletes participated in AIPP programs outside of the organized school sport. Inquiries on the number of student‐athletes participating in multiple sports or how many months per year of sport participation an athlete participated in were not included in this study.

## CONCLUSIONS

Despite widespread knowledge of AIPPs across high school athletic team coaching and training staff, only 3.5% of teams currently utilize these programs. Most common barriers to implementing these programs include a lack of trained staff and time restraints during training.

## DISCLOSURES

The authors (K.B.F., S.C., M.E.B.) declare the following financial interests/personal relationships which may be considered as potential competing interests: K.B.F. reports a relationship with Vericel that includes consulting or advisory and speaking and lecture fees; reports a relationship with DePuy Orthopaedics that includes consulting or advisory. S.C. reports a relationship with the American Orthopaedic Society for Sports Medicine that includes board membership; reports a relationship with Arthrex that includes nonfinancial support; reports a relationship with CONMED Linvatec that includes consulting or advisory; reports a relationship with the International Society of Arthroscopy Knee Surgery and Orthopaedic Sports Medicine that includes board membership; reports a relationship with Slack Incorporated that includes funding grants; reports a relationship with Zimmer that includes funding grants and speaking and lecture fees. M.E.B. reports a relationship with the *American Journal of Sports Medicine* that includes board membership; reports a relationship with the American Orthopaedic Society for Sports Medicine that includes board membership; reports a relationship with the *Video Journal of Sports Medicine* that includes board membership. The other authors (H.R.P., K.J., E.S., R.G.) declare that they have no known competing financial interests or personal relationships that could have appeared to influence the work reported in this article.
